# Melatonin Improves Oocyte Maturation and Mitochondrial Functions by Reducing Bisphenol A-Derived Superoxide in Porcine Oocytes In Vitro

**DOI:** 10.3390/ijms19113422

**Published:** 2018-10-31

**Authors:** Hyo-Jin Park, Soo-Yong Park, Jin-Woo Kim, Seul-Gi Yang, Min-Ji Kim, Ho-Guen Jegal, In-Su Kim, Young-Kug Choo, Deog-Bon Koo

**Affiliations:** 1 Department of Biotechnology, College of Engineering, Daegu University, 201 Daegudae-ro, Jillyang, Gyeongsan, Gyeongbuk 38453, Korea; wh10287@naver.com (H.-J.P.); clapdragon88@naver.com (S.-Y.P.); 38317kjw@hanmail.net (J.-W.K.); foreverday37@naver.com (S.-G.Y.); dldks2282@naver.com (M.-J.K.), lol4su@naver.com (H.-G.J.); insoo81422@naver.com (I.-S.K.); 2 Department of Biological Science, College of Natural Sciences, Wonkwang University, 460 Iksandae-ro, Iksan, Jeonbuk 54538, Korea

**Keywords:** bisphenol A, superoxide, Mito-TEMPO, melatonin, porcine oocyte maturation

## Abstract

Bisphenol A (BPA) is synthetic organic compound that exhibits estrogen-like properties and it induces mitochondrial superoxide production. Melatonin (Mela) protects against BPA-mediated cell damage and apoptosis. However, the antioxidative effects of Mela against BPA-induced superoxide production in porcine oocytes are still not known. In this study, we investigated the antioxidative effects of Mela against BPA-derived superoxide on oocyte maturation in pigs. To investigate the effects of the superoxide specific scavenger, Mito-TEMPO, on porcine oocyte maturation in response to BPA exposure apoptosis proteins, we treated the oocytes with Mito-TEMPO (0.1 µM) after pre-treating them with BPA (75 µM) for 22 h. As expected, the reduction in meiotic maturation and cumulus cell expansion of cumulus-oocyte-complexes (COCs) in the BPA (75 µM) treated group was recovered (*p* < 0.01) by treatment with Mito-TEMPO (0.1 µM). An increase in the levels of mitochondrial apoptotic proteins (AIF, cleaved Cas 3 and cleaved Parp1) in response to BPA-induced damage was also reduced by Mito-TEMPO treatment in porcine COCs. Interestingly, we confirmed the positive effects of Mela with respect to superoxide production upon BPA exposure during oocyte maturation and also confirmed the reduction in mitochondrial apoptosis in Mela (0.1 µM)-treated porcine COCs. These results provide evidence for the first time that antioxidative effects of Mela on BPA-derived superoxide improve porcine oocyte maturation.

## 1. Introduction

In vitro culture (IVC) system of porcine oocyte is commonly used to investigate the complex mechanism of female reproduction under IVC condition. Especially, the regulation mechanisms of reactive oxygen species (ROS) production and antioxidant enzymes in the regulation of important events of meiotic maturation and cumulus cells expansion during in vitro maturation (IVM) of porcine oocytes are already investigated. According to many studies, the mechanisms of oxidative stress and the protection against ROS play important roles in improving meiotic maturation and blastocyst formation during IVC, including oocyte maturation and pre-implantation embryo development [1,2,3]. According to a recent study, superoxide production occurs naturally as the intermediary products of cellular metabolism between cumulus cells and oocytes during the maturation process of cumulus-oocyte complexes (COCs) [4]. Moreover, ROS act as second signal molecules and modulate various cellular functions, such as meiotic cell cycle resumption, arrest, and mitochondrial-mediated apoptosis in oocytes of several mammalian species [5]. In addition, oxidative stress may cause cell death and it hinders nuclear and cytoplasmic maturation in oocytes [6]. A highly oxygenated environment induces ROS accumulation during in vitro embryo culture and lead to DNA damage impairing embryo development in porcine species [7].

Mitochondria is known as the core of the cellular energy metabolism, because it is the major site of ATP generation through mitochondrial oxidative phosphorylation [8]. During the synthesis of energy by mitochondria, superoxide is generated as a result of the reduction of O_2_ [9]. During oocyte maturation, COCs provide metabolic support as an energy source that is synthesized in the mitochondria [10]. In porcine oocytes, changes in mitochondrial distribution and mitochondria-specific antioxidant enzymes expression via increasing ROS production are involved in the meiotic maturation and cumulus cell expansion during pre-ovulatory maturation [11].

Bisphenol-A (BPA), a monomer of polycarbonate plastics, is an ingredient of epoxy and polystyrene resins and is widely used in consumer products such as in the coating of water pipe walls, food packaging, and plastic bottles [12]. BPA is an endocrine-disrupting chemical (EDC) that has been reported to accelerate estrogenic activity through binding to estrogen receptors. Also, BPA exposure affects the nervous system, as well as the secretion of female hypothalamic hormone [13,14]. BPA exposure has been reported to cause various defects in female reproduction functions, including in the inhibition of meiotic maturation [15] and embryonic development [16], in hormone production [17] and in female rat offspring. As well as, BPA induces apoptosis and cell cycle arrest in reproductive tract cancer cell lines [18] and prostate cancer cells [19,20]. In addition, BPA is commonly associated with oxidative stress, DNA damage, and apoptosis in porcine oocytes [21]. According to a recent study, BPA exposure increased the production of a reactive oxygen response (ROS) including superoxide from mitochondria, and induced mitochondrial dysfunction and apoptosis [22]. Notably, a previous report demonstrated that BPA exposure maintained high mitochondrial-derived ROS (Mito-ROS; superoxide anions) levels, and disturbed mitochondrial homeostasis in human HepG2 hepatoma cells [22]. Despite the fact that the negative effects of BPA exposure inducing oxidative stress on the female reproductive system have been well studied, virtually nothing is known about mitochondrial-specific ROS or superoxide production by BPA in porcine oocyte maturation and cumulus cell expansion in COCs. Mito-TEMPO (triphenylphosphonium chloride) is a specific scavenger of superoxide that is derived from mitochondria and a physiochemical compound mimicking superoxide dismutase [23]. Our previous study demonstrated the positive effects of Mito-TEMPO on porcine embryo development via the improvement of mitochondria functions [24]. However, the effects of ROS scavenger Mito-TEMPO on superoxide production from mitochondria after BPA exposure during porcine oocyte maturation are not yet well understood.

Many studies have also shown that melatonin (Mela) as a scavenging ROS or antioxidant plays an important role in improving porcine oocyte maturation, cumulus cell expansion of COCs, and embryo development [25,26]. In addition, oxidative stress impairs oocyte quality, while Mela protects oocytes from damage that is induced by free radicals. Previous reports also demonstrated that the positive effect of Mela on reducing oxidative stress from free radical damage improves oocyte maturation, fertilization rates, and embryonic development. For this reason, Mela is indispensable in porcine embryo in vitro cultures as an antioxidative factor [27]. Recently, Mela was reported to control cellular damages in response to BPA-induced superoxide [28]. The BPA-induced perturbation of mitochondrial marker enzymes in the testes of mice and its amelioration through Mela has been previously studied. Moreover, Mela was found to protect the testes and sperm quality against BPA-induced oxidative toxicity [28]. Mela ameliorates oxidative stress, modulates cellular death pathway proteins, and protects the rat cerebrum against BPA-derived oxidative stress. Many studies have previously reported on the protective effects of Mela against BPA-induced oxidative stress, ROS production, and cellular apoptosis. In this aspect, although the anti-oxidative effects of Mela on reducing of various ROS production have been reported in previous study [29], no direct information is available regarding the Mela antioxidant system response to BPA-induced oxidative stress on porcine oocyte maturation. Furthermore, protective mechanisms of Mito-TEMPO-like effects of melatonin against BPA-induced superoxide production in porcine oocyte maturation have not yet been reported.

Therefore, we hypothesized that Mela has an effect on the reduction of superoxide production upon BPA exposure, such as Mito-TEMPO via the regulation of mitochondrial functions or mitochondrial mediated apoptosis in porcine COCs during IVM. In the present study, we investigated the relationship between mitochondrial dysfunctions and poor oocyte quality upon BPA exposure, and whether the antioxidant Mela or Mito-TEMPO as a superoxide scavenger improves oocyte quality and maturation. The aim of this study was to confirm the protective role of Mela in the reduction of the superoxide production response as a result of BPA exposure during meiotic maturation and cumulus cell expansion in maturing porcine COCs.

## 2. Results

### 2.1. Effect of BPA Exposure on Meiotic Maturation and Cumulus cell Expansion in Porcine Oocytes

**Figure 1:**
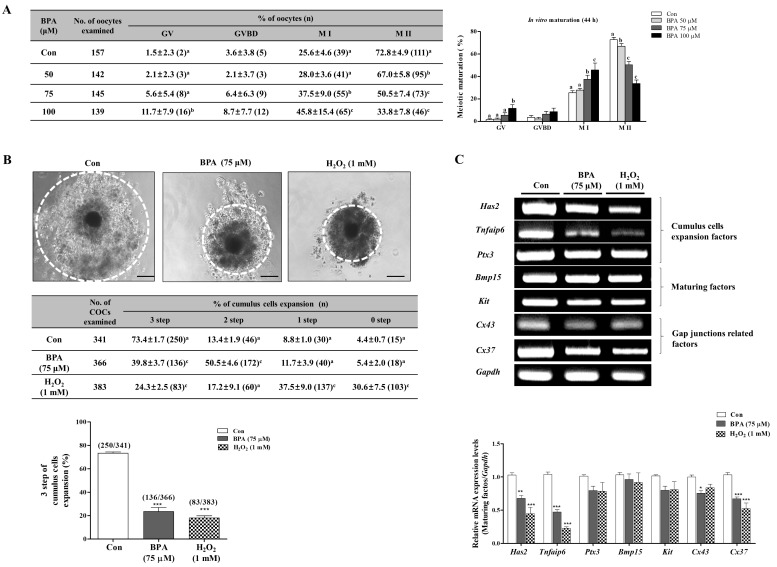
Investigation of meiotic maturation and cumulus cell expansion by BPA exposure in porcine oocyte maturation. Meiotic stages were classified as germinal vesicle (GV), germinal vesicle breakdown (GVBD), meiosis I (M I), and meiosis II (M II). (**A**) Diagram of porcine oocyte meiotic maturation by orcein staining according to BPA treatment concentrations (50, 75, and 100 µM) after 44 h of IVM. Summarized table of porcine oocyte meiotic maturation in pigs. Data are means ± SD. Different superscript letters denote a significant difference (*p* < 0.05). (**B**) Changes in cumulus cell expansion percentages in matured porcine cumulus-oocyte complexes (COCs) after BPA (75 μM) or H_2_O_2_ (0.1 mM)-treatment as described in Supplementary Figure S1. (**C**) The mRNA levels of cumulus cell expansion factors (*Has2, Tnfaip6* and *Ptx3*), maturing factors (*Bmp15* and *Kit*), and gap junction related factors (*Cx37* and *Cx43*) in maturing porcine cumulus oocyte complexes (COC) were measured using RT-PCR. Relative folds of genes were obtained by normalizing signals to *Gapdh*. Histogram values of densitometry analysis were obtained using ImageJ (NIH, MD) software. All experiments were carried out in triplicate and all values were presented as the mean ± standard error of the mean (S.E.M). Data were analyzed using one-way ANOVA followed by Bonferroni’s Multiple Comparison Test. Differences were considered significant at * *p* < 0.05, ** *p* < 0.01, *** *p* < 0.001 as compared to the control (Con) group. Scale bars = 200 µm.

Porcine oocytes matured in maturation medium supplemented with various concentrations (50, 75, and 100 µM) of BPA for 44 h. We then investigated the effects of different concentrations (50, 75, and 100 µM) of BPA on meiotic maturation by using orcein staining in porcine oocytes. As shown in Figure 1A, the meiotic maturation (M II stage) of porcine oocytes after 44 h of IVM significantly decreased according to the BPA concentrations compared to that in the control (Con) group (Con: 72.8 ± 4.9% vs. BPA 50 μM: 67.0 ± 5.8%; *p* < 0.05, 75 μM: 50.5 ± 7.4% and 100 μM: 33.8 ± 7.8%; *p* < 0.001). This result indicates that BPA has a negative effect on meiotic maturation in porcine oocytes after IVM. Based on this, we used 75 µM of BPA for subsequent experiments as the appropriate concentration.

In addition, for delineating the oxidative stress of BPA-induced ROS production on porcine oocyte maturation, we used a hydrogen peroxide (H_2_O_2_: 1 mM). Many studies have shown that the expansion of cumulus cells can be regulated by cumulus cell expansion factors (*Has2*: hyaluronan synthase2, *Tnfaip6*: tumor necrosis factor alpha-induced protein 6 *and Ptx3*: pentraxin3), maturing factors (*Bmp15*: bone morphogenetic protein 15 *and Kit*), and gap junction related factors (*Cx37*: connexin37 *and Cx43*: connexin43) [30,31]. Therefore, we investigated the genes that are related to cumulus cell expansion in porcine matured COCs after 44 h of IVM. As expected, compared to the matured COCs of the H_2_O_2_ 1 mM-treated group, cumulus cell expansion reduced significantly (*p* < 0.001) in the COCs of groups where BPA was 75 µM (Figure 1B). Additionally, *Has2*, *Tnfaip6 and Cx37* mRNA expression dramatically decreased (*p* < 0.001) in porcine COCs with 75 µM BPA. These results show that exposure to BPA (75 µM) hinders cumulus cell expansion in porcine COCs.

### 2.2. Measurement of BPA-Induced ROS Production, Mitochondrial Function, and Apoptosis in Porcine Matured COCs

**Figure 2:**
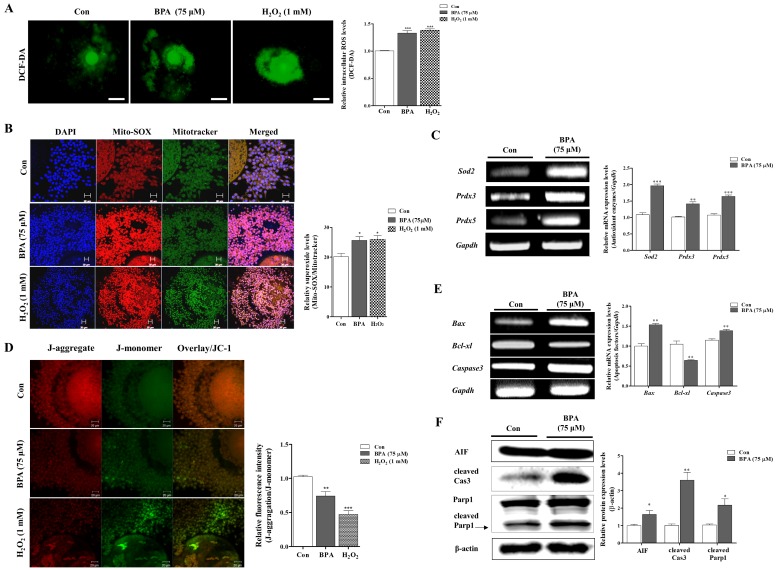
Changes in BPA-induced ROS production, mitochondrial dysfunction, and mitochondria-mediated apoptosis in matured porcine COCs. (**A**) Detection of intracellular ROS levels by using DCF-DA staining on porcine COCs after BPA (75 μM) or H_2_O_2_ (0.1 mM)-treatment, respectively. (**B**) Identification of mitochondria-specific superoxide by Mito-SOX staining in matured COCs after BPA (75 μM) and H_2_O_2_ (0.1 mM) treatment. COCs from BPA (75 µM)-treated group were stained with Mito-SOX (red fluorescence) and Mitotracker (green fluorescence) mitochondria detection dyes and observed via confocal microscopy (LSM700, Carl Zeiss, Germany). Scale bar = 20 µm. (**C**) The mRNA levels of mitochondria-related antioxidant enzymes (*Sod2*, *Prdx3* and *Prdx5*) on maturing porcine COCs were measured using RT-PCR. (**D**) Measurement of mitochondrial membrane potential (MMP) by JC-1 staining in matured COCs after BPA treatment. COCs from treated groups (Bisphenol-A (BPA) 75 μM or H_2_O_2_ 1 mM) were stained with JC-1 to evaluate MMP (Δψm) using confocal microscopy. Scale bar = 20 µm. (**E**) The mRNA levels of mitochondria-mediated apoptosis genes (*Bax, Bcl-xl* and *Caspase3*) were investigated in BPA and H_2_O_2_ treated COCs using RT-PCR, where in *Gapdh* was used as the internal control. (**F**) Western blotting results of AIF, cleaved Cas3 and cleaved Parp1 in porcine COCs compared to the control during porcine oocyte maturation. Relative folds of mitochondrial mediated apoptosis protein levels were obtained by normalizing the signals to β-actin. Histograms represent the values of densitometry analysis obtained using ImageJ software. Data in the bar graph are presented as the means ± SEM of three independent experiments (per 30 COCs). Differences were considered to be significant at * *p* < 0.05, ** *p* < 0.01, *** *p* < 0.001 compared to the control group.

Previous studies have found that BPA exposure induces mitochondrial-specific ROS and oxidative stress [22,23]. Therefore, we investigated the intracellular and mitochondrial ROS production by DCF-DA and Mito-SOX staining in porcine COCs after treatment with 75 μM BPA after 44 h of IVM. Intracellular ROS levels significantly increased in the BPA and H_2_O_2_ 1 mM treated-groups as compared to the control (*p* < 0.001; Figure 2A). Interestingly, the red fluorescence of Mito-SOX as a mitochondrial ROS specific detection dye increased (*p* < 0.05) in BPA-treated porcine COCs (Figure 2B). To confirm that ROS production was a result of BPA exposure during porcine oocyte maturation, we also examined the mRNA expression levels of various antioxidant enzymes including *glutathione peroxidase1 (Gpx1)*, *catalase, superoxide dismutase 1 (Sod1)*, *superoxide dismutase 2 (Sod2)*, and *peroxiredoxins (Prdxs)* through RT-PCR analysis. As shown in Supplementary Figure S3 and Figure 2C, the mRNA levels of mitochondria-related antioxidant enzymes (*Sod2, Prdx3,* and *Prdx5*) significantly increased (*p* < 0.001) in porcine COCs as a result of BPA (75 μM) exposure.

In the following experiment, we assessed the alterations in the mitochondrial membrane potential (MMP; Δψm) of porcine COCs after BPA treatment. BPA- or H_2_O_2_-treated COCs were stained with JC-1 (Figure 2D). As a result, a reduction in MMP was found in BPA-treated COCs. To identify the change in mitochondrial-mediated apoptosis factors, we performed RT-PCR analysis of *Bax*, *Bcl-xl*, and *Caspase3* mRNA expression and western blotting analysis of AIF, cleaved Cas3 and cleaved Parp1 in porcine COCs after BPA treatment. As expected, as shown in Figure 2E,F, the mRNA levels of *Bax and Caspase3* significantly increased (*p* < 0.01), and the protein levels of AIF, cleaved Cas3 and cleaved Parp1 also increased in the BPA treated group as compared to the control (*p* < 0.05). Thus, these results demonstrate that BPA-induced ROS production increased mitochondrial superoxide, mitochondrial dysfunction, and apoptosis in porcine COCs.

### 2.3. BPA-Induced ROS Production or Mitochondrial Damage in Porcine COCs Is Recovered by Mito-TEMPO, a Specific Superoxide Scavenger

**Figure 3:**
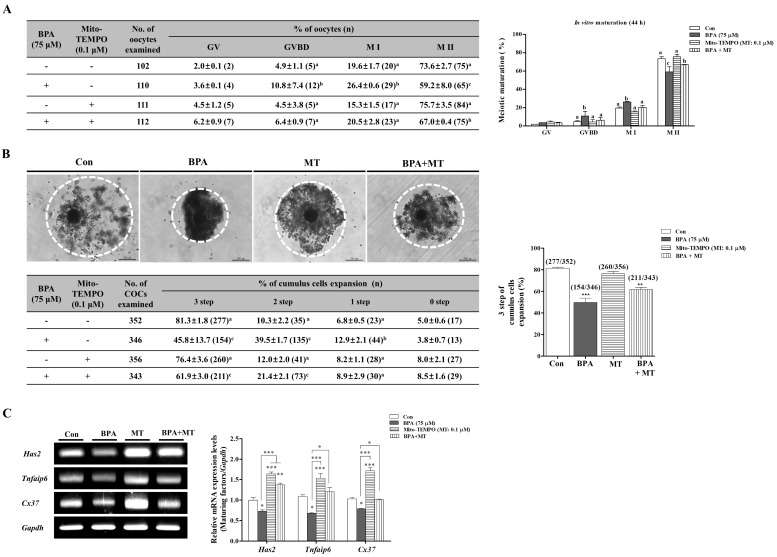
Investigation of meiotic maturation and cumulus cell expansion by Mito-TEMPO response to BPA-induced oxidative stress during porcine oocytes maturation. Meiotic stages were classified as GV, GVBD, M I, and M II. (**A**) Diagram of oocyte meiotic maturation after orcein staining of porcine oocytes in only BPA 75 μM, only Mito-TEMPO (MT; 0.1 μM) and MT treatment after BPA pre-treatment (BPA + MT) groups, respectively. Summarized table of porcine oocyte meiotic maturation. Data are means ± SD. Different superscript letters denote a significant difference (*p* < 0.05). (**B**) We divided the four groups (0, 1, 2, and 3 step) of COCs according to cumulus cells expansion at 44 h after IVM of porcine oocyte, as described in Supplementary Figure S2. Changes in cumulus cell expansion percentages in matured porcine COCs of BPA- and/or MT-treated groups. (**C**) The mRNA levels of cumulus cell expansion factors (*Has* and *Tnfaip6*) and gap junction related factors (*Cx37*) in maturing porcine COCs were measured by RT-PCR analysis. Relative folds of these genes were obtained by normalizing the signals to *Gapdh*. Histograms represent the values of densitometry analysis obtained by using ImageJ software. Data in the bar graph are presented as the means ± SEM of three independent experiments (per 30 COCs). Differences were considered to be significant at * *p* < 0.05, ** *p* < 0.01, *** *p* < 0.001 compared to the control group. Scale bar = 200 µm.

In our previous study, the use of Mito-TEMPO (MT; 0.1 μM) as a H_2_O_2_ or superoxide target scavenger improved blastocyst development through mitochondrial functions in porcine embryos [24]. To confirm the positive effects of MT in response to BPA-induced oxidative stress and mitochondrial dysfunctions during porcine oocyte maturation, we investigated meiotic maturation though 0.1 μM MT treatment alone (MT) and MT 0.1 μM treatment after BPA pre-treatment (BPA + MT) for 0–22 h IVM. As shown in Figure 3A, the reduction of meiotic maturation after BPA exposure was recovered in porcine COCs in NCSU medium supplemented with MT 0.1 μM. However, the meiotic maturation of only MT 0.1 μM treated porcine oocytes showed no change as compared to those in the other group (Con: 73.6 ± 2.7% vs. only BPA 75 μM: 59.2 ± 8.0%; *p* < 0.01, only MT 0.1 μM: 75.7 ± 3.5% and BPA+MT: 67.0 ± 0.4%; *p* < 0.05). In addition, the reduction of cumulus cells expansion as a result of BPA exposure in porcine COCs was also recovered in the MT 0.1 µM-treated group when compared to the control (*p* < 0.05; Figure 3B). Thus, the positive effects of the MT response to BPA exposure showed that the mRNA expression levels of *Has2*, *Tnfaip6*, and *Cx37* as cumulus cell expansion and gap junction related genes during oocyte maturation were re-decreased (Figure 3C).

**Figure 4:**
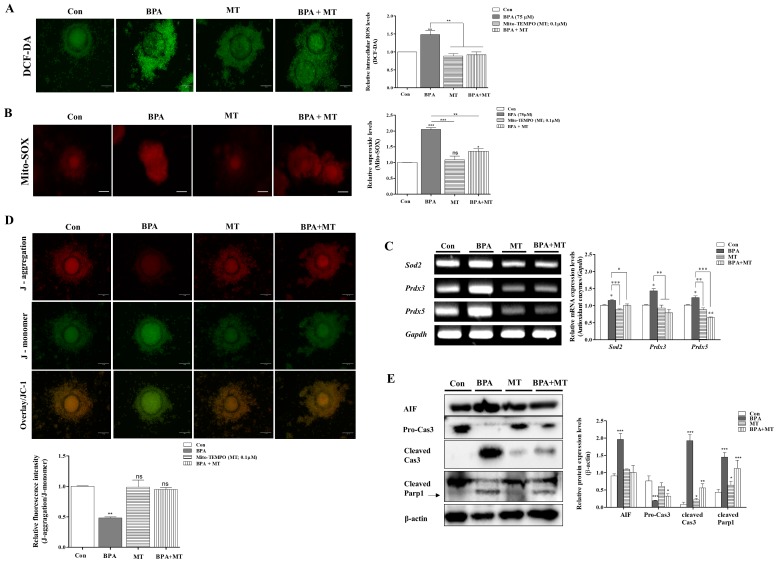
Positive effects of Mito-TEMPO (MT) response to BPA-induced ROS production, mitochondria dysfunction, and mitochondria-mediated apoptosis in mature porcine COCs. (**A**) Detection of intracellular ROS levels using DCF-DA staining in porcine COCs after BPA (75 μM) and/or MT (0.1 μM) treatment, respectively. (**B**) Identification of mitochondria-specific superoxide by Mito-SOX staining in matured COCs of BPA- and/or MT-treated groups. COCs from the treated groups were stained with Mito-SOX (red fluorescence) using the iRiS™ Digital Cell Image System (Logos Biosystems, Gyeonggi-do, Korea). Scale bar = 100 µm. (**C**) The mRNA levels of mitochondria-related antioxidant enzymes (*Sod2*, *Prdx3*, and *Prdx5*) in maturing porcine COCs from BPA-and/or MT-treated groups were measured using RT-PCR. (**D**) Measurement of MMP by JC-1 staining in matured COCs after BPA and/or MT treatment. COCs from treated groups were stained with JC-1 to evaluate MMP (Δψm) using the iRiS™ Digital Cell Image System (Korea). Scale bar = 100 µm. (**E**) Western blotting results of AIF, Pro-Cas3, cleaved Cas3 and cleaved Parp1 in BPA- and/or MT-treated porcine COCs as compared to the control. Relative folds of mitochondria-mediated apoptosis protein levels were obtained by normalizing the signals to β-actin. Histograms represent the values of densitometry analysis obtained using ImageJ software. Data in the bar graph are presented as the means ± SEM of three independent experiments (per 30 COCs). Differences were considered to be significant at * *p* < 0.05, ** *p* < 0.01, ***; *p* < 0.001 compared to control group.

Additionally, we performed a recovery experiment against BPA-induced mitochondrial ROS production and mitochondrial dysfunction by using MT 0.1 µM on porcine COCs. When compared to the only BPA-treated COCs after 44 h of IVM, intracellular ROS levels and superoxide production recovered significantly (*p* < 0.01) in the group where MT (0.1 µM) was added after BPA 75 μM pre-treatment (Figure 4A,B). The recovery effects after the addition of MT also increased the MMP, the mRNA levels of mitochondria-related antioxidant enzymes (*Sod2*, *Prdx3*, and *Prdx5*), and the protein levels of AIF, Pro-Cas3, cleaved Cas3, and Cleaved Parp1 in porcine COCs after 44 h of IVM (Figure 4C–E).

These results demonstrate that the protective effects of MT for the regulation of BPA-induced mitochondria ROS production and mitochondrial damages are required to maintain meiotic maturation and cumulus cells expansion during the IVM progression of porcine oocytes.

### 2.4. Protective Mito-TEMPO-Like Effects of Melatonin Against BPA-Induced ROS Production and Mitochondrial Damage in Porcine COCs

**Figure 5:**
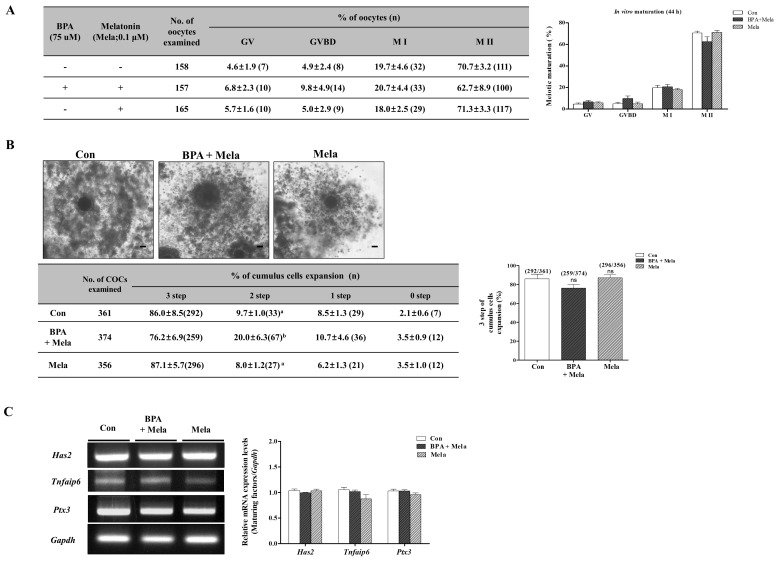
Effects of melatonin on meiotic maturation and cumulus cell expansion against BPA-induced oxidative stress during porcine oocyte maturation. Meiotic stages were classified as GV, GVBD, M I, and M II. (**A**) Diagram of oocytes meiotic maturation after orcein staining in porcine oocytes of only BPA 75 μM, only melatonin (Mela; 0.1 μM) and Mela treatment after BPA pre-treatment (BPA + Mela) groups, respectively. Summarized table of porcine oocyte meiotic maturation. Data are means ± SD. Different superscript letters denote a significant difference (*p* < 0.05). (**B**) Changes in cumulus cell expansion percentages in matured porcine COCs of BPA- and/or Mela-treated groups. (**C**) The mRNA levels of cumulus cells expansion factors (*Has*, *Tnfaip6*, and *Ptx3*) in porcine maturing COCs after BPA and/or Mela treatment were measured using RT-PCR. Relative folds of the genes were obtained by normalizing the signals to *Gapdh*. Histograms represent the values of densitometry analysis obtained using ImageJ software. Data in the bar graph are presented as the means ± SEM of three independent experiments (per 30 COCs). Scale bar = 200 µm.

Our previous study showed that melatonin (Mela) 0.1 μM treatment improves meiotic maturation and cumulus cell expansion in porcine oocytes after 44 h of IVM [32]. Therefore, we investigated whether the change during meiotic maturation are regulated by Mela 0.1 μM as a ROS scavenger under BPA-induced oxidative stress during porcine oocyte maturation. To analyze the relationship between the anti-oxidative effects of Mela and BPA-induced ROS production in porcine oocytes after 44 h of IVM, We carried out orcein staining, as shown in Figure 5A. We used BPA as a mitochondrial ROS inducer and Mela as an ROS scavenger. The porcine COCs of the Mela 0.1 µM treated group showed a recovery in terms of oocyte maturing damages with reduced meiotic maturation, reduced cumulus cell expansion, and overexpression of *Has2*, *Tnfaip6*, and *Ptx3* mRNA levels in BPA pre-treated COCs for 0–22 h of IVM I (Figure 5A–C). When compared to the only Mela-treated group, porcine oocyte maturation showed no changes in the control group.

**Figure 6:**
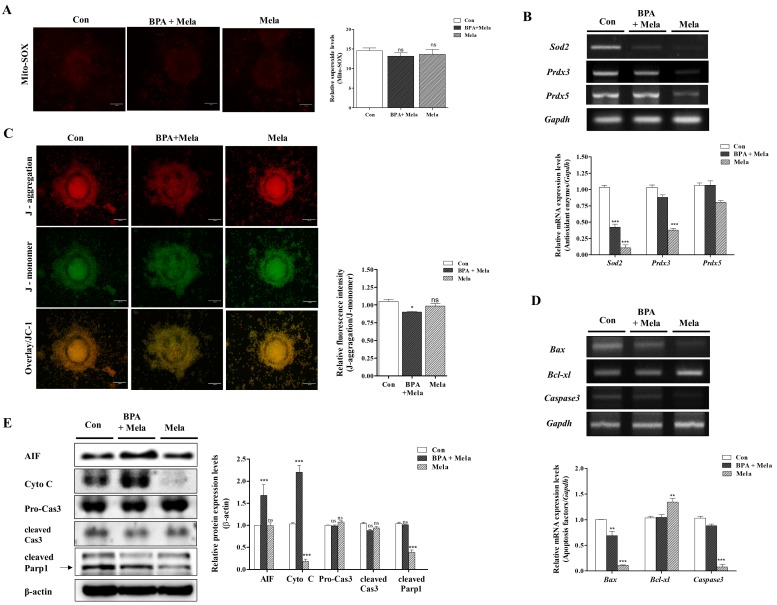
Protective effects of Mito-TEMPO-like Mela response to BPA-induced ROS production, mitochondrial dysfunction, and mitochondria-mediated apoptosis in mature porcine COCs. Detection of intracellular ROS levels using DCF-DA staining in porcine COCs after BPA (75 μM) and/or Mela (0.1 μM) treatment, respectively. (**A**) Identification of mitochondria-specific superoxide by Mito-SOX staining in matured COCs of BPA and/or Mela treatment groups. COCs from the treated groups were stained with Mito-SOX (red fluorescence) and Mitotracker (green fluorescence) as a mitochondria detection dyes using the iRiS™ Digital Cell Image System (Korea). Scale bar = 20 µm. (**B**) The mRNA levels of mitochondria-related antioxidant enzymes (*Sod2*, *Prdx3*, and *Prdx5*) in maturing porcine COCs after BPA and/or Mela treatment were measured using RT-PCR. (**C**) Measurement of MMP by JC-1 staining in matured COCs after BPA and/or Mela treatment. COCs from the treated groups were stained with JC-1 to evaluate MMP (Δψm) using the iRiS™ Digital Cell Image System (Korea). Scale bar = 20 µm. (**D**) The mRNA levels of mitochondrial mediated apoptosis genes (*Bax*, *Bcl-xl*, and *Caspase3*) were investigated in BPA- and Mela-treated COCs using RT-PCR, where *Gapdh* was used as the internal control. (**E**) Western blotting results of AIF, Cyto C, Pro-Cas3, cleaved Cas3, and cleaved Parp1 in BPA- and/or Mela-treated porcine COCs as compared to the control. Relative folds of mitochondria-mediated apoptosis protein levels were obtained by normalizing the signals to β-actin. Histograms represent the values of densitometry analysis obtained using ImageJ software. Data in the bar graph are presented as the means ± SEM of three independent experiments (per 30 COCs). Differences were considered significant at * *p* < 0.05, ** *p* < 0.01, *** *p* < 0.001 compared to the control group (ns = not significant).

Mitochondrial specific ROS production and MMP were recovered in the group where Mela (0.1 µM) was added after pre-treatment with BPA (75 µM) (Figure 6A,C). The addition of Mela, acting as superoxide scavenger with MT-like recovery effects, also significantly decreased the mRNA levels of *Sod2*, *Prdx3*, and *Prdx5* expression in porcine COCs (Figure 6B). Moreover, Mela in porcine COCs that were pre-treated with BPA decreased the expression level of cleaved Cas3 and cleaved Parp1 as well as mitochondrial-mediated apoptosis after 44 h of IVM (*p* < 0.001, Figure 6E). The increased *Bax* and *Caspase3* mRNA levels due to BPA exposure were reduced in Mela-treated COCs after 22–44 h of IVM (Figure 6D). These results demonstrate that the protective effects of Mela on BPA-induced ROS production and oxidative stress through pre-treatment for IVM I recovered oocyte maturation, cumulus cell expansion, antioxidant enzymes expression, dysfunction, and apoptosis from mitochondria in porcine COCs.

## 3. Discussion

In the present study, we investigated whether BPA-induced oxidative stress or mitochondrial-specific ROS (mito-ROS) production induced a reduction in meiotic maturation and cumulus cell expansion in porcine COCs after 44 h of IVM. As a result of BPA exposure, we observed that BPA-derived oxidative stress induced mitochondrial dysfunction and apoptosis signaling from mitochondria in porcine COCs. In addition, our present study also suggests that Mito-TEMPO (MT) acts as a mitochondrial-specific ROS scavenger against BPA-induced ROS production via regulating mitochondrial functions and mitochondrial-mediated apoptosis during porcine oocyte maturation. We are the first to show the protective effects of melatonin (Mela), which, in similar way to the superoxide scavenger MT, reduces BPA-induced mito-ROS production. Moreover, as a protection factor from BPA-induced oxidative stress, Mela regulates the expression of antioxidant enzymes (*Sod2*, *Prdx3*, and *Prdx5*), mitochondrial dysfunctions, and mitochondria mediated apoptosis during porcine oocyte maturation.

It is well known that BPA-induced oxidative stress lead to serious inhibitions in meiotic maturation, cumulus cells expansion, maturing factor secretion, and the maintenance of oocyte maturation in pigs [15]. Therefore, as shown in Figure 1A, low meiotic maturation was found as a result of BPA treatment at different concentration (50, 75, and 100 μM) in porcine COCs. Despite the need for proper ROS generation and cellular apoptosis conditions for the in vitro maturation of porcine COCs [33], the expansion of cumulus cells from maturing COCs was significantly reduced after 75 μM BPA and 1 mM H_2_O_2_ treatments (Figure 1B). However, BPA treated COCs showed a 30% reduction in meiotic maturation as compared to control, and two-step of cumulus cell expansion was higher than in the H_2_O_2_ treated COCs in pigs. Previous studies have demonstrated that cumulus cell expansion is involved in the production of the extracellular matrix (ECM) [34]. Hyaluronan comprises the major structural backbone of the matrix and it is synthesized by hyaluronan synthase 2 (*Has2*). Tumor necrosis factor alpha-induced protein 6 (*Tnfaip6*) and pentraxin3 (*Ptx3*) are also important components of the ECM [35]. The interaction between oocyte and granulosa cells is necessary for bidirectional communication. Communication of oocyte-granulosa cells occurs via gap junctions [36]. Gap junctions are also involved in the transfer of molecules between oocytes and cumulus cells. The communication between cumulus cells and oocyte-secreted factors, such as *Bmp15* and *Kit* is also fostered by regulating folliculogenesis [37]. The mRNA levels of *Has2* and *Tnfaip6* as COCs secretion factors and the gap junction related gene, *Cx37*, significantly decreased (*p* < 0.001) in porcine COCs of the BPA treated group (Figure 1C). BPA inhibited cumulus cell expansion in porcine COCs through the mRNA expression of COCs. Based on these results, we determined that treatment with 75 μM of BPA induced ROS production and oxidative damage. Therefore, this concentration was used in subsequent experiments.

To confirm whether there was a loss in porcine oocyte maturation, cumulus cell expansion, and mitochondrial function as a result of superoxide production after BPA exposure, we identified the functional link between mitochondrial function and antioxidant enzymes from mitochondria and mitochondrial mediated apoptotic factors (AIF, cleaved Cas3, and cleaved Parp1) in BPA treated porcine COCs (Figure 2). As shown in Figure 2B,C, BPA treated porcine COCs showed increased superoxide production and reduction in mitochondria related antioxidant enzyme levels (*Sod2, Prdx3,* and *Prdx5*). During the IVM of porcine oocytes, H_2_O_2_ or superoxide from mitochondria was produced [38]. In addition, porcine oocyte maturation requires appropriate ROS production from mitochondria during IVM progression [33]. Changes in the expression of various antioxidant enzymes such as *Glutathione peroxidase1 (Gpx1)*, *Superoxide dismutase* (*Sod1* and *Sod2*), *Catalase*, and *Peroxiredoxins (Prdxs)* suggest that oxidative stress affects oocyte maturation in pigs [21]. In other words, ROS generation is an invariable phenomenon in the in vitro culture of porcine oocytes or embryos, and it may be possible that an appropriate level of ROS production by anti-oxidant enzymes could positively influences oocyte maturation in vitro [39]. Although various antioxidant enzymes play essential roles in the porcine oocyte maturation and cumulus cells expansion of COCs [6,40], no direct information is available regarding mitochondria-specific ROS-related antioxidant systems resulting from BPA-induced oxidative stress. In addition, as shown in Figure 2D, we confirmed BPA-induced mitochondrial dysfunction through a decreased in MMP in porcine COCs. Many studies reported that BPA induces cellular or mitochondrial mediated apoptosis activation [41,42]. Based on the results presented in Figure 2, we concluded that BPA-induced mitochondrial ROS and mitochondrial dysfunction, led to mitochondrial-mediated apoptosis in mature porcine COCs.

Mito-TEMPO as a superoxide-specific scavenger is known to decrease mitochondrial ROS and inhibit total cellular ROS, including H_2_O_2_ [21]. However, the effect of Mito-TEMPO on BPA-induced oxidative stress during porcine oocyte maturation has not yet been elucidated. As shown in Figure 4, we investigated whether BPA-induced ROS production and oxidative stress in regulation of mitochondrial functions by MMP maintenance in porcine COCs was recovered by Mito-TEMPO as a specific superoxide scavenger. These observations provide the first evidence regarding the recovery mechanisms and the protective effects provided by Mito-TEMPO against BPA-induced oxidative stress or damages during IVM in porcine COCs. In addition, the reduction of mitochondrial ROS by Mito-TEMPO may play an important role in mitochondria-related mechanisms and apoptosis signaling during porcine oocytes maturation.

Many previous studies have been showed that the roles of melatonin in respect of antioxidative effects against hydrogen peroxide (H_2_O_2_)-induced oxidative stress [43,44,45]. Additionally, BPA is induced the H_2_O_2_ [46], superoxide production [47], and oxidative stress-derived cell death [48]. Above all, several studies have demonstrated a relationship between the production of ROS from BPA exposure and the anti-oxidative effects of Mela [12,28,49]. Our previous study showed that Mela reduces the ER stress response to ROS production involved in porcine oocyte maturation [32]. We also showed that Mito-TEMPO acts as a superoxide scavenger to improve blastocyst development in porcine embryos [24]. Mela is known for its positive effects against BPA-induced oxidative stress and ROS production, as shown in previous studies [28,49].

Recently, Mela was reported to reduce oxidative stress and mitochondrial apoptosis signaling resulting from exposure to BPA [50]. Mela was also found to ameliorate BPA-induced DNA damage in the germ cells of rats [12], and it protects oocyte quality from BPA-induced deterioration in mice [51]. Additionally, Mela suppresses BPA-induced ROS production and significantly compromises mitochondrial function [49]. As shown in Figure 5, the reduction of meiotic maturation, cumulus cell expansion, and cumulus cells secretion factors (*Has2*, *Tnfaip6*, and *Ptx3*) by BPA exposure was upregulated in porcine COCs of Mela-treatment groups. As a result, as shown in Figure 6B, an increase in mitochondria-related antioxidant enzyme (*Sod2*, *Prdx3*, and *Prdx5*) expression by BPA was recovered in Mela-treated porcine COCs. Therefore, we confirmed that Mela has an antioxidative effect and it protects against the overexpression of three antioxidant enzymes resulting from BPA-induced oxidative stress and toxicity in porcine COCs. In addition, Mela treatment result in a reduction in MMP and mitochondria apoptosis from BPA-induced oxidative stress in porcine COCs (Figure 6).

**Figure 7:**
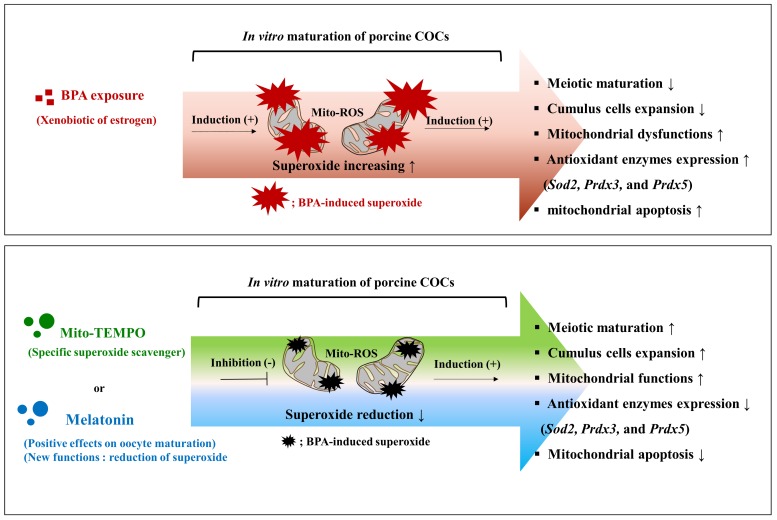
Role of Mito-TEMPO-like melatonin in oocyte maturation, cumulus cells expansion, maintenance of mitochondrial functions, and mitochondrial apoptosis detects that are induced by BPA oxidative stress during porcine oocyte maturation. Graphical summary. Top panel: Mitochondrial ROS production and oxidative stress due to BPA exposure during porcine oocyte maturation resulted in decreased meiotic maturation and cumulus cell expansion, reduced mitochondria function, and increased mitochondria-mediated apoptosis in maturing porcine COCs. Bottom panel: Deficiencies during porcine oocyte maturation due to BPA-induced oxidative stress after 44 h of IVM, were recovered by the protective effects of Mito-TEMPO-like melatonin.

Consequentially, BPA-induced ROS production plays an important role in mitochondrial function and apoptosis during meiotic maturation and cumulus cell expansion in the IVM progression of porcine COCs. Moreover, Mito-TEMPO-like effects of melatonin acts as a superoxide scavenger to reduce BPA-induced ROS production during porcine oocyte maturation and both mitochondrial function and apoptosis are regulated by reducing superoxide production in Mito-TEMPO or melatonin treated COCs in pigs (Figure 7).

## 4. Materials and Methods

### 4.1. Chemicals

Unless otherwise stated, all chemicals that were used in this study were purchased from Sigma Chemical Co. (St. Louis, MO, USA).

### 4.2. In Vitro Maturation (IVM)

Porcine ovaries were obtained from six months old of female pigs (Yorkshire/Landrace (♀) × Duroc (♂), 100 kg) at the local abattoirs (Gyeongsan and Daegu City) and they transported to the laboratory in 0.9% saline (*w*/*v*) with 75 μg/mL potassium penicillin G at around 30–35 °C. Immature cumulus-oocyte complexes (COCs) were aspirated from follicles (3–6 mm in diameter) using a 10 mL syringe with an 18-gauge needle. The undamaged COCs with a similar cytoplasm quality and surrounding cumulus cells were selected by mouth pipetting and were then washed three times in Tyrode’s lactate-*N*-2-hydroxyethylpiperazine-ethanesulfonic acid (TL-HEPES) medium. About 50–60 immature COCs were matured in 500 µL of in vitro maturation (IVM) medium at 38.5 °C with 5% CO_2_. Bovine serum albumin (BSA) free North Carolina State University (NCSU)-23 medium supplemented with 10% follicular fluid (*v*/*v*), 0.57 mM cysteine, 10 ng/mL β-mercaptoethanol, 10 ng/mL EGF, 10 IU/mL pregnant mare’s serum gonadotropin (PMSG), and 10 IU/mL human chorionic gonadotropin (hCG) was used for oocyte maturation [52]. After culturing for 22 h (IVM I), the same media was used for oocyte maturation without PMSG/hCG for 22–44 h (IVM II). During the maturation period, Bisphenol A (BPA) (concentration of 50, 75, and 100 µM) and H_2_O_2_ (1 mM) were added to the maturation medium NCSU-23 for 44 h for inducing oxidative stress. Then, either Mito-TEMPO (MT; 0.1 µM) or melatonin (Mela; 0.1 μM) for IVM II was added for the reduction of BPA-induced mitochondrial ROS treated with NCSU-23 after BPA pre-treatment for 22 h of IVM (Supplementary Figure S1).

### 4.3. Assessments of Cumulus Cell Expansion and Acetic-Orcein Staining

Cumulus expansion of porcine oocytes was evaluated under a microscope (Leica, Solms, Germany) after 44 h of IVM. We divided the cumulus cell expansion of porcine COCs after 44 h of IVM into three steps. This experimental method has been previously been described [15] (Supplementary Figure S2). After 44 h of IVM, meiotic maturations were distinguished according to the nuclear stages. Oocytes denuded by pipetting in TL-HEPES medium containing 0.1% hyaluronidase were rinsed with polyvinyl alcohol (PVA)-PBS and were mounted on microscope slides. The samples were fixed for three days in acetic acid/ethanol (1:3, *v*/*v*) and were stained with 1% acetic-orcein (*v*/*v*) for 5 min. The meiotic stage of the samples was evaluated under a microscope (Leica, Solms, Germany).

### 4.4. RNA Extraction and Reverse Transcription (RT)—PCR

Total RNA was isolated using the Trizol reagent (Invitrogen, Carlsbad, CA, USA) according to the manufacturer’s instructions. RNA concentration and purity were measured with an OPTIZEN NanoQ (Mecasys, Daejeon, Korea). Subsequently, 1 µg/µL of total RNA and AccuPower^®^ RT-PCR Premix (Bioneer Inc., Daejeon, Korea) was used to synthesize cDNA. Specific primers for the sequences of interest (Table 1, Table 2 and Table 3) were designed using the NCBI database. PCR was carried out at 95 °C for 5 min, followed by 30–35 cycles comprising the following steps: 95 °C for 30 s, 55–61 °C for 30 s, 72 °C for 30 s, and 72 °C for 5 min. The PCR products were separated by electrophoresis on a 2% agarose gel, stained with ethidium bromide (EtBr), and photographed under UV illumination. Band intensities were quantified while using the ImageJ software (version 1.47, National Institutes of Health, Bethesda, MD, USA).

### 4.5. Staining of DCF-DA, Mito-SOX and Mitotracker

The levels of intracellular ROS in porcine COCs were measured using the dichlorofluorescein diacetate method (DCF-DA; Molecular Probes, Eugene, OR, USA), as described previously [53]. After washing three times in PBS-polyvinylalcohol (PVA) medium, mature COCs were transferred into IVM II medium containing 5 μM DCF-DA and incubated for 20 min at 38.5 °C, 5% CO_2_. After the COCs were washed three times with 0.1% PVA in PBS, the fluorescent emissions from the COCs were recorded using an Olympus DP 70 camera (Olympus, Tokyo, Japan). Mito-SOX (red) and the Mitotracker (green)-positive stained cytoplasm were examined using a Cal Zeiss LSM 700 confocal microscope (Carl Zeiss, Thornwood, Germany). Porcine COCs were washed three times in 0.1% PVA-PBS (*v*/*v*) and then cultured in 300 μL of IVM II medium mixed Mito-SOX (500:1) (Invitrogen, Carlsbad, CA, USA) and Mitotracker (250:1) (Invitrogen) at 37 °C for 30 min. Afterwards, COCs were fixed in 2.5% glutaraldehyde/PBS (*v*/*v*) for 1 h. For membrane permeabilization, the fixed embryos were permeabilized using 0.1% Triton X-100 (*v*/*v*) for 30 min, then stained with Hoechst 33,342 (2 mg/mL) in the medium, and covered with a cover glass after mounting on glass slides with a mounting solution.

### 4.6. Measurement of Mitochondrial Membrane Potential (MMP)

Mature COCs after 44 h of IVM, were washed three times in 0.1% PVA-PBS (*v*/*v*), and incubated in IVM II medium containing JC-1 kit (100:1) (Cyman Chemical, Ann Arbor, MI, USA) in a four-well dish at 37 °C, for 30 min. Next, COCs were washed three times in 0.1% PVA-PBS (*v*/*v*) and fixed in 2.5% glutaraldehyde/PBS (*v*/*v*) solution for 1 h. MMP was assessed according to the changes in color, from green to red, as Δψm increased, previously described [54]. Finally, COCs were examined using a Cal Zeiss LSM 700 confocal microscope (Carl Zeiss, Thornwood, Germany).

### 4.7. Protein Extraction and Western Blot Analysis

Maturated COC (30 per sample) lysates were extracted in PRO-PREP protein lysis buffer (iNtRON Biotechnology, Seongnam, South Korea). The COC lysates were separated while using SDS-PAGE in 12% gels. After electrophoresis, the separated proteins were transferred onto nitrocellulose membranes (Pall Life Sciences, Port Washington, NY, USA). After blocking, the membranes were incubated with anti-cleaved Cas3 (1:3000; #9664; Cell Signaling Technology, Danvers, MA, USA), anti-Parp1 (1:4000; SC-7150; Santa Cruz, CA, USA), anti-AIF (1:3000; SC-390619; Santa Cruz), anti-Cyto chrom C (Cyto C; 1:3000; Ab90529; Abcam, Cambridge, MA, USA), anti-Pro-Cas3 (1:3000; SC-1225; Santa Cruz) and anti-β-actin (1:3000; SC-47778; Santa Cruz, CA, USA) antibodies. The membranes were then incubated with a secondary HRP-conjugated anti-mouse/rabbit IgG (Thermo, Rockford, IL, USA) and an anti-goat IgG (Abfrontier, Seoul, South Korea) secondary antibody overnight. Antibody binding was detected using an ECL kit (Advansta, Menlo Park, CA, USA), according to the manufacturer’s instructions. The amount of protein based on the band densities was measured using Fusion Solo software (ver. 16.08, Vilber Lourmat, France), and the bands were scanned using ImageJ.

### 4.8. Statistical Analysis

All percentage data and data sets were subjected to arcsine transformation and expressed as the mean ± standard deviation (SD). All of the values from the western blot and RT-PCR experiments were presented as the mean ± standard error of the mean (SEM). The results were analyzed using one-way ANOVA followed by Bonferroni’s and Tukey’s multiple comparison test and *t*-tests. Histogram values of densitometry analysis were obtained using ImageJ (NIH, Bethesda, MD, USA). All data were obtained using GraphPad Prism 5.0 (San Diego, CA, USA). Differences were considered significant at * *p* < 0.05, ** *p* < 0.01, and *** *p* < 0.001.

## 5. Conclusions

Our results provided first evidence that melatonin improves the meiotic maturation and cumulus cells expansion via the reducing BPA-induced superoxide production or oxidative stress and mitochondrial-derived apoptosis during IVM of porcine oocyte. Based on these results, this study suggests that Mela has protective effects that are similar to Mito-TEMPO against ROS production and oxidative stress from exposure to BPA in porcine COCs. Therefore, the present study demonstrates a link between the protective effects of Mela in response to BPA-induced oxidative stress and porcine oocyte maturation via the regulation of mitochondrial ROS generation and mitochondria mechanisms.

## Figures and Tables

**Table 1:** Primer sequences of secretion factor genes from matured COCs for RT-PCR.

Genes	Primer Sequences	T_m_ °C	Gene Bank Accession No.	Base Pairs
*Has2*	F(5′–3′): TGGCTGTACAATGCGATGTG R(5′–3′): TGGGTGGTGTGATTTTCACC	55	(NM_214053.1)	402
*Tnfaip6*	F(5′–3′): TCTTCCTGTGGGAAGAGGCT R(5′–3′): GTCCGTCTGAACAGAAGCGA	55	(NM_001159607.1)	337
*Ptx3*	F(5′–3′): TCAGTGCCTGCATTTGGGTC R(5′–3′): TTCTGAACAAGGGCATGTAG	58	(NM_001244783.1)	225
*Bmp15*	F(5′–3′): GGATCCAGAAAAGCACAACC R(5′–3′): GCATTTCATCCCTGGACACT	55	(NM_001005155.1)	227
*Kit*	F(5′–3′): TGTATTCACAGAGACTTGGCGG R(5′–3′): TTACGTGGTCAAAGGAAACG	55	(NM_001044525.1)	124
*Cx43*	F(5′–3′): ACTGAGCCCCTCCAAAGACT R(5′–3′): GGCTAATTACAGTGCCGAGC	55	(NM_001244212.1)	191
*Cx37*	F(5′–3′): TTCCTTGAGAAGCTGCTGGA R(5′–3′): CATCTCCCACATCCGCTACT	55	(NM_001244224.1)	217
*Gapdh*	F(5′–3′): TCGGAGTGAACGGATTTC R(5′–3′): CCTGGAAGATGGTGATGG	53.7	(NM_001206359.1)	230

*Has2*; *HA synthase 2*, *Tnfaip6*; *TNF-a-induced protein 6*, *Ptx3*; *Pentraxin 3*, *Bmp15*; *Bone Morphogenetic Protein 15*, *Cx43*; *Connexins 43*, *Cx37*; *Connexins 37*, *Gapdh*; *Glyceraldehyde-3-Phosphate Dehydrogenase*, *Tm*; *Meting Temperature*, *F*; *Forward*, *R*; *Reverse*.

**Table 2:** Primer sequences of antioxidant enzyme genes in porcine for RT-PCR.

Genes	Primer Sequences	T_m_ °C	Gene Bank Accession No.	Base Pairs
*Gpx1*	F(5′–3′): CACAACGGTGCGGGACTA R(5′–3′): GTCTCCAGTGTGTCGCAATG	**54**	(NM_214201.1)	326
*Catalase*	F(5′–3′): CGAAGGCGAAGGTGTTTG R(5′–3′): GGATATGGATCGCACACT	**50**	(NM_214301.2)	374
*Sod1*	F(5′–3′): GCCAAAGGATCAAGAGAGGC R(5′–3′): GTCGTTTGGCCTGTGGTGTA	**55**	(NM_001190422.1)	226
*Sod2*	F(5′–3′): GCAGCTCGAGCAGGAATCTGG R(5′–3′): ACGCGGCCTACGTGAACAA	**59.7**	(NM_214127.2)	163
*Prdx1*	F(5′–3′): AGAAGCAAGGAGGACTGGGA R(5′–3′): GCCTGATGTCCAGAAGAGCA	**55**	(XM_003128042.3)	300
*Prdx2*	F(5′–3′): CACCTGGCTTGGATCAACAC R(5′–3′): TCCAGGCCTTCCAGTACACA	**55**	(NM_001244474.1)	249
*Prdx3*	F(5′–3′): AGTGGATTCCCACTTCAGCC R(5′–3′): AACCCATGGAGAAGTCTGCC	**55.1**	(NM_001244531.1)	290
*Prdx4*	F(5′–3′): AGTTTACCCATCTGGCCTGG R(5′–3′): GTCCTGCTGGTTGGAAACCT	**55**	(XM_005673497.2)	296
*Prdx5*	F(5’–3’): ACCTTCCAGGGTTTGTGGAG R(5′–3′): CCTGAATGTGGAGCCAGATG	**55**	(NM_214144.1)	285
*Prdx6*	F(5′–3′): ATGCCTGTGACAGCTCGTGT R(5′-3′): ACCAAAGAGCTCCCATCTGG	**55.2**	(NM_214408.1)	263
*Gapdh*	F(5′–3′): TCGGAGTGAACGGATTTC R(5′–3′): CCTGGAAGATGGTGATGG	**53.7**	(NM_001206359.1)	230

*Gpx1; Glutathione peroxidase 1, Sod1; Superoxide dismutase 1, Sod2; Superoxide dismutase 2, Prdx1; Peroxiredoxin 1, Prdx2; Peroxiredoxin 2, Prdx3; Peroxiredoxin 3, Prdx4; Peroxiredoxin 4, Prdx5; Peroxiredoxin 5, Prdx6; Peroxiredoxin 6, Tm; melting temperature.*

**Table 3:** Primer sequences of mitochondrial mediated apoptosis factors for RT-PCR.

Genes	Primer Sequences	T_m_ °C	Gene Bank Accession No.	Base Pairs
*Bax*	F(5′–3′): AAGCGCATTGGAGATGAACT R(5′–3′): CTGGACTTCCTTCGAGATCG	**50**	(XM_003127290.4)	251
*Bcl-xl*	F(5′–3′): AGGGCATTCAGTGACCTGAC R(5′–3′): CACCTAGAGCCTTGGATCCA	**55**	(NM_214285.1)	242
*Caspase3*	F(5′–3′): GAGGCAGACTTCTTGTATGC R(5′–3′): TTCCATGTATTGTGTCCATGC	**50**	(NM_214131.1)	238
*Gapdh*	F(5′–3′): TCGGAGTGAACGGATTTC R(5′–3′): CCTGGAAGATGGTGATGG	**53.7**	(NM_001206359.1)	230

*Bax*; *Bcl2*-associated X protein, *Bcl-xl*; B-cell lymphoma-extra large.

## Supplementary Materials

Supplementary Materials can be found at http://www.mdpi.com/1422-0067/19/11/3422/s1.
