# Supplementary material for: Melatonin Improves Oocyte Maturation and Mitochondrial Functions by Reducing Bisphenol A-Derived Superoxide in Porcine Oocytes In Vitro
Source: Int J Mol Sci. 2018 Oct 31;19(11):3422. doi: 10.3390/ijms19113422 (PMC6274783; doi:10.3390/ijms19113422)
Supplement: Supplementary file 1 [file ijms-19-03422-s001.pdf]

International Journal of
Molecular Sciences

MDPI

Supplementary Materials:

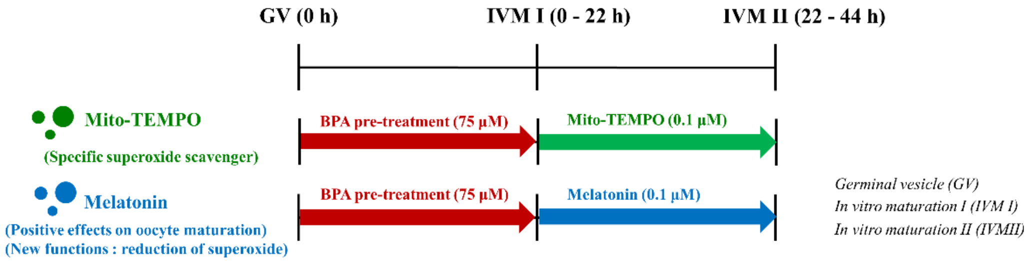

Supplementary Fig. 1 Graphical description of Mito-TEMPO or melatonin treatment in porcine oocyte maturation

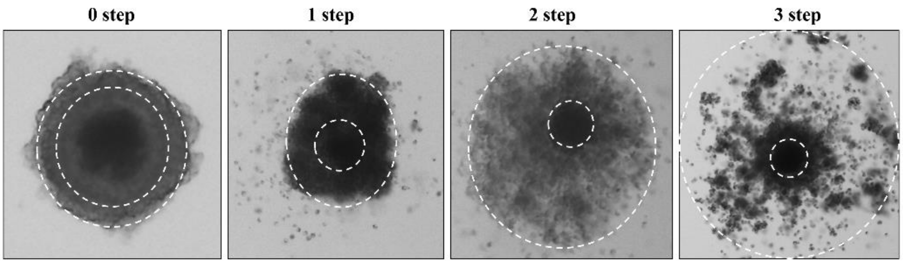

Supplementary Fig. 2 Division of cumulus cells expansion in porcine COCs at 44 h of IVM.

The degree of expansion was assessed according to a subjective scoring system from 0 to 3 steps as follows: 0 step is no expansion, 1 step is separation of only the outermost layer of cumulus cell, 2 step is further expansion involving the outer half of the cumulus oophorus, 3 step is further expansion up to, complete expansion including the corona radiate cells.

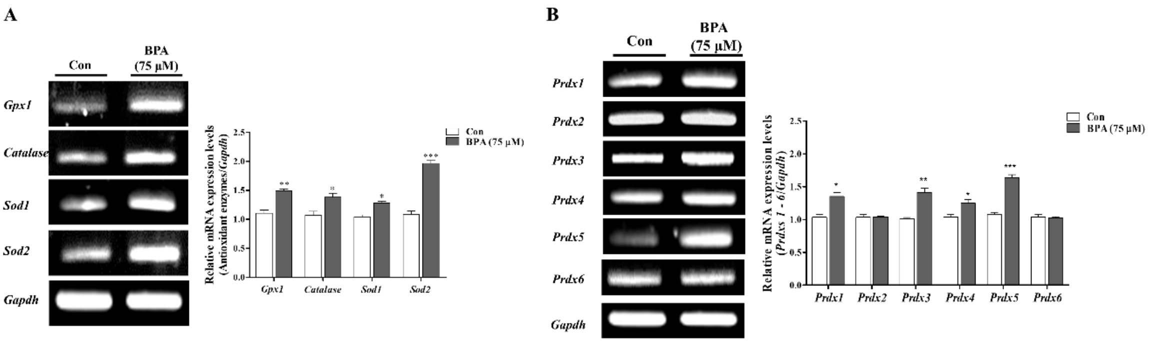

Supplementary Fig. 3 Changes in antioxidant enzymes in maturing COCs after BPA treatment during porcine oocyte maturation. (A and B) The mRNA levels of antioxidant enzymes (A; Gpx1, Catalase, Sod1 and Sod2, B; *Prdx1, Prdx2, Prdx3, Prdx4, Prdx5 and Prdx6*) in porcine maturing COCs after BPA 75 μM treatment were measured using RT-PCR. Relative folds of the genes were obtained by normalizing the signals to *Gapdh*. Histograms represent the values of densitometry analysis obtained using ImageJ software. Data in the bar graph are presented as the means ± SEM of three independent experiments (per 30 COCs). Differences were considered significant at * $p < 0.05$, ** $p < 0.01$, ***; $p < 0.001$ compared to the control group.
